# ATPase-Independent Type-III Protein Secretion in *Salmonella enterica*

**DOI:** 10.1371/journal.pgen.1004800

**Published:** 2014-11-13

**Authors:** Marc Erhardt, Max E. Mertens, Florian D. Fabiani, Kelly T. Hughes

**Affiliations:** 1 Junior Research Group Infection Biology of Salmonella, Helmholtz Centre for Infection Research, Braunschweig, Germany; 2 Department of Biology, University of Utah, Salt Lake City, Utah, United States of America; Universidad de Sevilla, Spain

## Abstract

Type-III protein secretion systems are utilized by gram-negative pathogens to secrete building blocks of the bacterial flagellum, virulence effectors from the cytoplasm into host cells, and structural subunits of the needle complex. The flagellar type-III secretion apparatus utilizes both the energy of the proton motive force and ATP hydrolysis to energize substrate unfolding and translocation. We report formation of functional flagella in the absence of type-III ATPase activity by mutations that increased the proton motive force and flagellar substrate levels. We additionally show that increased proton motive force bypassed the requirement of the *Salmonella* pathogenicity island 1 virulence-associated type-III ATPase for secretion. Our data support a role for type-III ATPases in enhancing secretion efficiency under limited secretion substrate concentrations and reveal the dispensability of ATPase activity in the type-III protein export process.

## Author Summary

The type-III protein secretion apparatus is a complex nanomachine responsible for secretion of building blocks and substrate proteins of the flagellum and the virulence-associated injectisome needle complex of many gram-negative pathogens. Type-III secretion systems utilize the energy of the proton motive force and ATP hydrolysis of a cytoplasmic ATPase to drive substrate export. The cytoplasmic components of the secretion system share strong homology to the F_o_F_1_ ATP synthase and it is thought that the flagellum was derived from a proto F_o_F_1_-ATP synthase where ATP hydrolysis energized the export process. Here, we report the dispensability of ATPase activity for the type-III protein export process in *Salmonella*. This finding has important implications for the evolution of the bacterial flagellum and type-III secretion systems, suggesting that a proto ATPase was added to a primordial proton-powered type-III export system with the evolutionary benefit of facilitating the export process.

## Introduction

**Figure 1:**
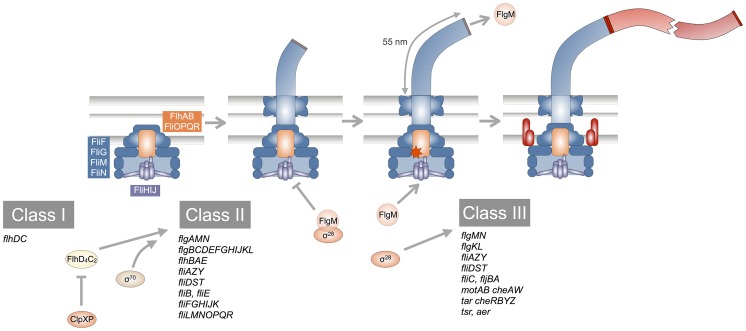
Schematic overview of the flagellar transcriptional hierarchy and biogenesis. The flagellar transcriptional hierarchy of *Salmonella enterica* is composed of three classes of promoters. The Class I promoter transcribes a single operon encoding for the master regulator of the flagellar transcriptional hierarchy, the FlhD_4_C_2_ complex, which is negatively regulated by ClpXP protease. FlhD_4_C_2_, together with σ^70^, directs RNA polymerase to transcribe from Class II promoters. Genes transcribed from Class II promoters encode structural components of the hook-basal-body complex (shaded in blue), the flagellar type-III secretion apparatus (composed of the membrane proteins FlhA, FlhB, FliO, FliP, FliQ and FliR; and the soluble proteins FliH, FliI and FliJ), as well as regulatory proteins, in particular the flagellar-specific σ-factor, σ^28^ (encoded by *fliA*), and its cognate anti-σ factor, FlgM. The hook-basal-body is completed as soon as the hook reaches an approximate length of 55 nm, upon which the type-III secretion apparatus switches secretion specificity to its late-substrate secretion mode (indicated by the orange star). Subsequently, the late substrate FlgM is exported out of the cell, thereby freeing σ^28^ to turn on transcription from Class III promoters. Class III gene products include the filament subunits, motor-force generators and the chemotactic system (shaded in red).

Many bacteria move by rotating a rigid, helical organelle, the flagellum [1]. The flagellum represents one of the smallest motor complexes known and enables bacteria to move through liquids (swimming) [2] and highly viscous environments or surfaces (swarming) [3]. In addition to the chemotactic behavior, flagellar motility contributes to bacterial pathogenesis by promoting bacteria-host interactions, adherence, biofilm formation and invasion of eukaryotic cells [4]. A schematic overview of flagellar biogenesis in *Salmonella enterica* is shown in Figure 1. Export of the building blocks of the flagellum is mediated by a flagellar-specific type-III secretion system (fT3SS), whose core cytoplasmic and inner-membrane export apparatus components (FliHIJ FliPQR FlhAB) are evolutionarily and functionally related to the virulence-associated type-III secretion systems (vT3SS) of pathogenic Gram-negative bacteria [5].

The core export apparatus components of both fT3SS and vT3SS mediate the translocation of proteins across the inner membrane and exhibit stringent substrate recognition and high speed of protein translocation [6]. Core export apparatus assembly initiates in the cytoplasmic membrane with the stepwise addition of FliF, FliG, FliM and FliN to produce a structure resembling a cytoplasmic-facing cup [7]. The inner membrane export apparatus (export gate) of the fT3SS assembles within the cup-like structure and consists of the integral membrane proteins FlhB, FlhA, FliO, FliP, FliQ and FliR [8]. Functionally associated with the fT3SS is a cytoplasmic ATPase complex composed of the FliI, FliH and FliJ proteins [6]. The ATPase FliI forms a hexameric ring-shaped structure [9] and together with the negative regulator FliH [10] regulates the initial entry of substrates into the export gate by ensuring that FliI-dependent ATP hydrolysis occurs in concert with secretion. FliH interacts with the N-terminal region of FliI and prevents its ATPase activity until the export apparatus is competent to utilize ATP hydrolysis in the export process [10], [11]. Interestingly, FliI and FliJ show remarkable structural similarity to the α/β and γ-subunits of F_O_F_1_ ATPases, respectively [12], [13]. FliH is homologous to the stalk subunits b and δ of F_O_F_1_ ATPases [14]. Until recently, it was presumed that ATP hydrolysis was the energy source for type-III secretion. However, it was demonstrated that elimination of the proton motive force (PMF, consisting of the protein gradient ΔpH and the membrane potential Δψ) inhibited flagellar type-III secretion [15], [16]. ATP hydrolysis might energize chaperone release and secretion substrate unfolding [17]. Further functions of the FliHIJ proteins might be in localizing the ATPase/secretion substrate complexes to the vicinity of the export gate by interaction with the switch-complex protein FliN [18], [19] and the cytoplasmic domain of FlhA [12], [20]. An efficient interaction of FliJ with FlhA requires support of FliH and FliI upon which FliJ switches the conformation of the export gate from a low-efficient proton-protein antiporter to a highly efficient, Δψ-driven export system. FliJ is thus essential for the Δψ-driven type-III protein export and in the absence of FliH and FliI, the type-III export apparatus requires both Δψ and ΔpH of the PMF to couple the energy derived from the proton influx with substrate protein secretion [21]. Additional described functions of FliJ include chaperone-like activities by preventing premature aggregation of both early and late secretion substrates [22], as well as a function in substrate selectivity as a chaperone escort protein that specifically recruits unladen substrate-specific chaperones FlgN and FliT during assembly of the filament junction and cap substructures [23]. FliJ is also implicated in facilitating FliI hexamer ring formation, thereby stimulating the ATPase activity [12], [23].

Similar to the fT3SS, the vT3SS is an essential component of the virulence-associated needle complexes encoded on *Salmonella* Pathogenicity Islands 1 and 2 (Spi1 and Spi2) [5], [24]. The ATPase associated with the Spi1 vT3SS is encoded by *invC* [25]. InvC functions in effector substrate recognition and induces chaperone release and subsequent unfolding of the respective secretion substrate in an ATP-dependent manner [17].

Type-III protein secretion is a process of great complexity and the importance of ATP hydrolysis for the type-III secretion process was unclear. In the present work, we demonstrate that type-III protein secretion can efficiently occur in the absence of ATPase activity in both flagellar and virulence-associated T3SS.

## Results

We demonstrate that flagellar ATPase activity is not essential for secretion by the isolation and characterization of spontaneous suppressor mutants that assemble flagella in a Δ*fliHI* genetic background. These motile revertants mapped to four locations on the *Salmonella* chromosome. One of these linkage groups included the *flh* flagellar region that likely included suppressors similar to the *flhA* and *flhB* mutations described before [16], [26] and were not characterized further. Based on the chromosomal location of the remaining three linkage groups, we identified candidates for genes that could account for the motile suppression phenotypes. One linkage group included the *clpXP* operon. Loss of ClpXP protease stabilizes the flagellar master regulatory complex, FlhDC [27], upregulating flagellar gene expression. It was previously shown that transposon insertions in the *clpP* gene increased flagellar secretion substrate levels sufficiently to overcome the requirement for the cytoplasmic C-ring for T3SS-dependent substrate translocation across the inner membrane [28]. The third group of motile revertants were linked to the ATP synthase structural genes (*atp*) (Fig. S1). ATP synthesis, flagellum assembly and flagellar rotation are all dependent on the PMF and it has been previously described that a null mutation in *atp* increases the PMF [29], [30]. Increased PMF could thus restore proper flagellar assembly and function if the fT3SS is primarily PMF-dependent. Consistently, Martinez-Argudo et al. [31] reported that mutations in *atpB* overcame the inhibitory effect of the unspecific type-III inhibitors salicylidene acylhydrazides on flagellar motility. The final linkage group was located to a set of flagellar genes that included a negative regulatory gene (*flgM*) of the flagellar-specific transcription factor σ^28^. Strains defective in genes required for flagellar hook-basal body assembly (including *fliH*, *fliI* and *fliJ*) accumulate the FlgM anti-σ^28^ factor in the cytoplasm, which prevents σ^28^-dependent flagellar class 3 promoter expression. Thus, loss of FlgM could account for suppression of the motility defect of the Δ*fliHI* allele.

**Figure 2:**
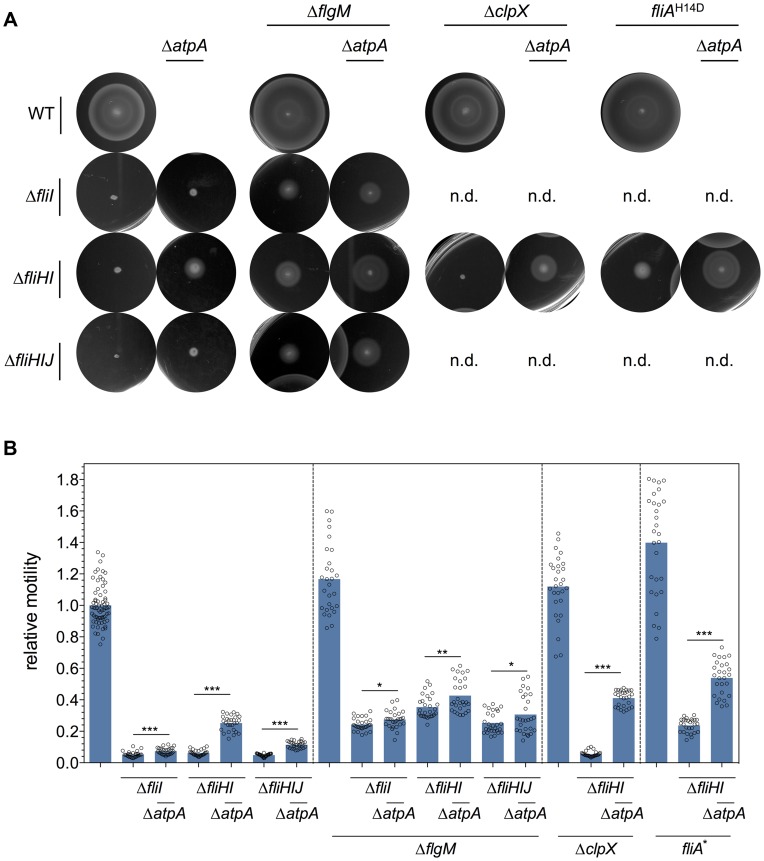
Motility of *fliHIJ* mutants is increased by mutations in Δ*atpA*, *ΔflgM*, *ΔclpX* and *fliA* ^H14D^. Null mutations in *atpA*, *flgM*, *clpX*, and the more stable FliA^H14D^ variant increased motility of *fliHIJ* mutant strains in a swimming motility assay using 0.3% soft agar plates. (**A**) Representative soft agar motility plates after 4.5 hours incubation at 37°C of the FliC-phase locked wildtype and *fliHIJ* mutant strains. n.d., not determined. (**B**) Quantified relative motility of *fliHIJ* mutant strains. The diameter of the motility swarm relative to the wildtype was measured after 4.5 hours incubation. Biological replicates are shown as individual data points. Data were analyzed by the Student's *t* test. Stars indicate significantly different motility (*, P<0.05; **, P<0.01; ***, P<0.001).

We constructed deletion mutants of *clpX* and *atpA* and tested the suppressor function in various *fliHIJ* deletion backgrounds by monitoring their swimming behavior in soft agar plates (Fig. 2A and Fig. S2). The absence of the F_O_F_1_ ATPase restored motility of a *fliI* deletion mutant to about 5% of the wildtype, of a *fliHI* deletion mutant to about 25% of the wildtype and of a *fliHIJ* deletion mutant to about 11% of the wildtype (Fig. 2B). In an otherwise wildtype background, deletion of *atpA* resulted in a pronounced growth defect (Fig. S3), however did not affect the free-swimming velocity (Fig. S4).

As noted above, the absence of ClpXP protease components affects flagellar class 2 and class 3 gene expression by preventing proteolytic degradation of the flagellar master regulatory protein complex FlhDC. Similarly, a deletion of the FlgM anti-σ^28^ factor, or a FlgM-resistant σ^28^ mutant (FliA^H14D^) results in an increase of flagellar class 3 gene expression. We thus analyzed the motility of clean deletion mutants of *clpX* and *flgM*, and the *fliA* ^H15D^ allele. When combined with deletions of the *fliHIJ* ATPase complex components, the *flgM* deletion and the FlgM-resistant σ^28^ mutant raised motility levels up to 24–35% of the wildtype. The *clpX* deletion, however, had no apparent effect on motility when deleted alone. Next, the *flgM*, *clpX*, and *fliA* ^H15D^ mutations were combined with a null mutation of the F_O_F_1_ ATPase as described above. Interestingly, the *atpA* mutation had only a slight effect on the motility of *fliI*, *fliHI* and *fliHIJ* mutants in the *flgM* null background, which could be explained by impaired coordination of flagellar Class 3 gene expression with the assembly state of the flagellum. However, the apparent PMF increase of the *atpA* mutation did substantially increase motility in the *clpX* null and *fliA* ^H15D^ backgrounds to approximately 41–54% of the wildtype (Fig. 2B and Fig. S2). We thus concluded that a 20% increase in PMF by inactivation of the ATP synthase [29], [30] substantially enhances fT3SS export capability and thereby bypasses the FliHIJ ATPase requirement for flagellar function. We next reasoned that an artificial induction of an additional PMF-draining system would antagonize the suppressor function of the *atpA* null strain in the FliHIJ ATPase mutant backgrounds, which we tested by inducing the PMF-dependent inner membrane tetracycline/proton antiporter (TetA). Induction of the TetA efflux pump counteracted the suppressor phenotype of the *atpA* deletion of various *fliHIJ* deletion mutants in a motility assay (Fig. S5). We conclude that high levels of TetA tetracycline/proton antiporter activity drained the PMF and thereby negatively affected protein export via the fT3SS.

**Figure 3:**
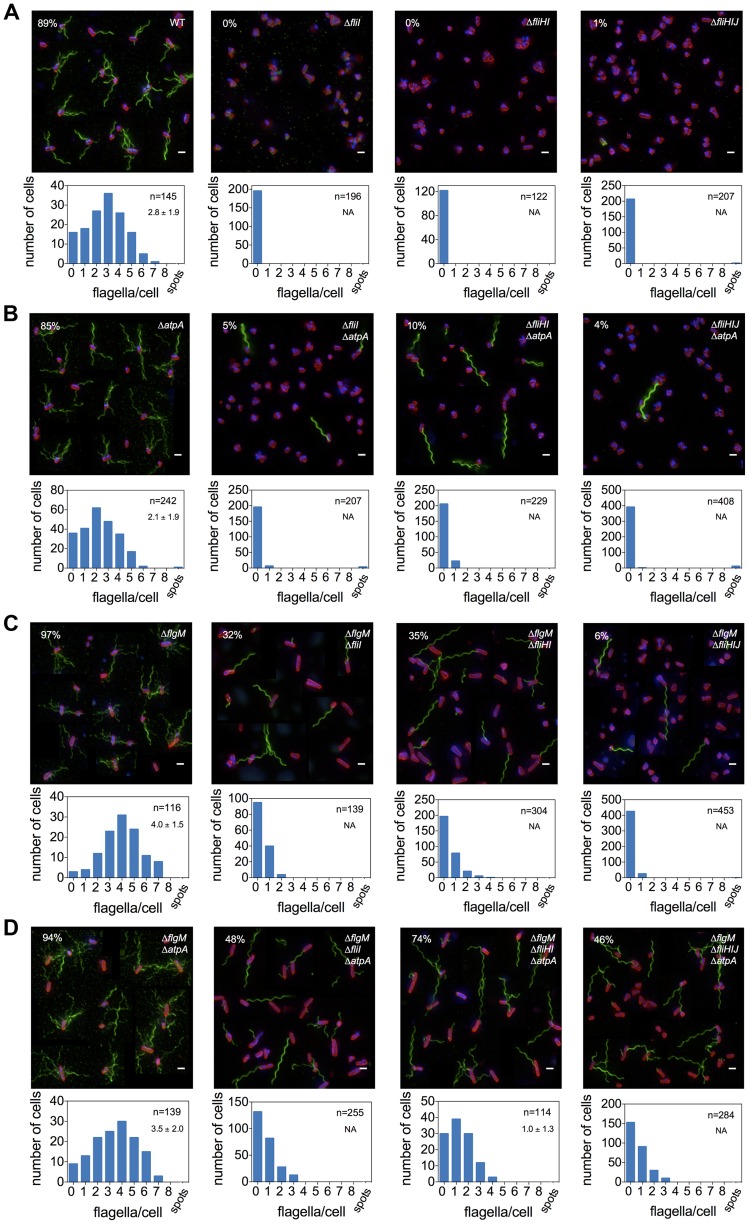
Frequency of flagellar filament formation of *fliHIJ* mutants is increased in *atpA* and *flgM* null backgrounds. The absence of the flagellar ATPase subunits FliH, FliI and FliJ results in a non-flagellated phenotype (**A**). Additional deletions in *atpA* (**B**) and *flgM* (**C**) substantially increase the frequency of flagellar filament formation of *fliI*, *fliHI* and *fliHIJ* mutant strains. Flagellar formation in the *flgM* null background is further enhanced by combination with the *atpA* mutation (**D**). Top: A montage of representative fluorescent microscopy images is shown. Flagellar filaments were stained using anti-FliC immunostaining and detected by FITC-coupled secondary antibodies (green), DNA was stained using Hoechst (blue) and cell membranes using FM-64 (red). Scale bar 2 µm. The percentage of cells with at least one filament is presented in the upper left corner. Bottom: Histogram of counted flagellar filaments per cell body. Number of counted cells and average number of filaments per cell +/− standard deviation based on Gaussian non-linear regression analysis is given in the upper right hand corner.

The type-III secretion apparatus is a PMF-powered protein exporter and we reasoned that increased PMF due to the *atpA* deletion provided additional energy to overcome the requirement of the flagellar ATPase complex for flagellar assembly. Flagellar filament assembly will only occur in the presence of a functional and efficiently working fT3SS. Deletion mutants of *fliI*, *fliHI* and *fliHIJ* were non-flagellated (Fig. 3A). The *atpA* deletion resulted in a small increase in the frequency of bacteria that produced functional flagella in a *fliI* and *fliHIJ* deletion strain up to approximately 4% of the population. However, the absence of *atpA* in a *fliHI* deletion mutant increased the frequency of flagella formation to about 10% of the bacteria (Fig. 3B). There appears to exist an important bottleneck in flagellar assembly that was overcome by the increase in PMF in the *atpA* deletion mutants. We observed formation of only one, singular filament in cases where flagellar assembly could proceed beyond completion of a hook-basal body complex. It has been shown previously that under wildtype conditions about 90% of detectable HBBs had a filament attached. Thus, virtually every secretion competent HBB had switched to late substrate secretion mode [32]. Since we observed only single flagellar filaments of wildtype lengths in our *fliHI* mutants (Fig. 3B and Fig. S6), it is a possibility that a localization mechanism exists that preferentially targets late flagellar substrates to a secretion system that is in late-type secretion mode.

**Figure 4:**
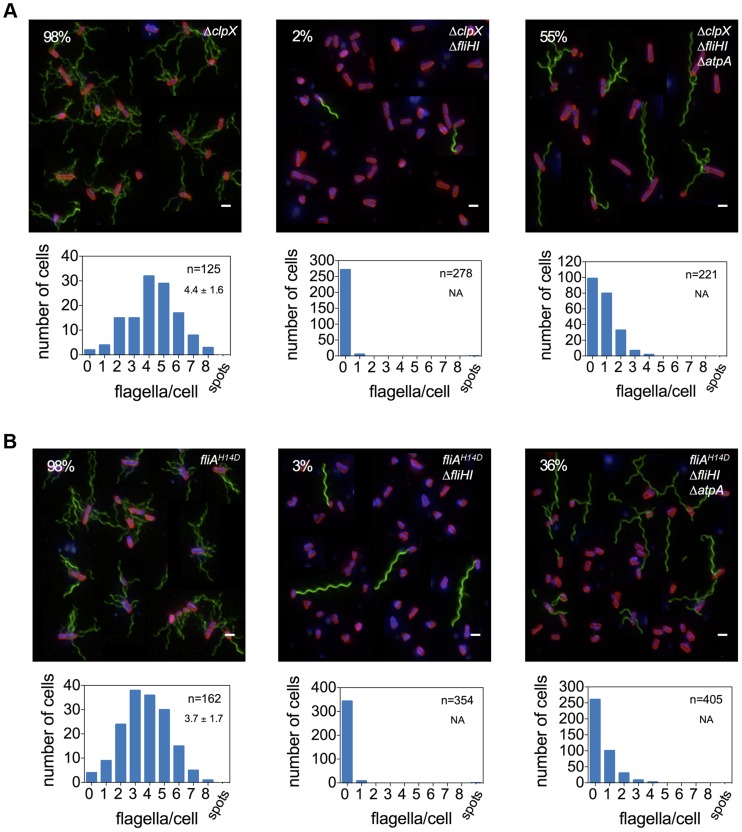
Frequency of flagellar filament formation of a *fliHI* mutant strain is increased in *clpX* null and *fliA^H14D^* backgrounds. A deletion in *clpX* (**A**) and the more stable *fliA^H14D^* variant (**B**) increase the frequency of flagellar filament formation in a *fliHI* mutant strain. Flagellar formation is further enhanced by combination with an *atpA* mutation. Top: A montage of representative fluorescent microscopy images is shown. Flagellar filaments were stained using anti-FliC immunostaining and detected by FITC-coupled secondary antibodies (green), DNA was stained using Hoechst (blue) and cell membranes using FM-64 (red). Scale bar 2 µm. The percentage of cells with at least one filament is presented in the upper left corner. Bottom: Histogram of counted flagellar filaments per cell body. Number of counted cells and average number of filaments per cell +/− standard deviation based on Gaussian non-linear regression analysis is given in the upper right hand corner.

**Figure 5:**
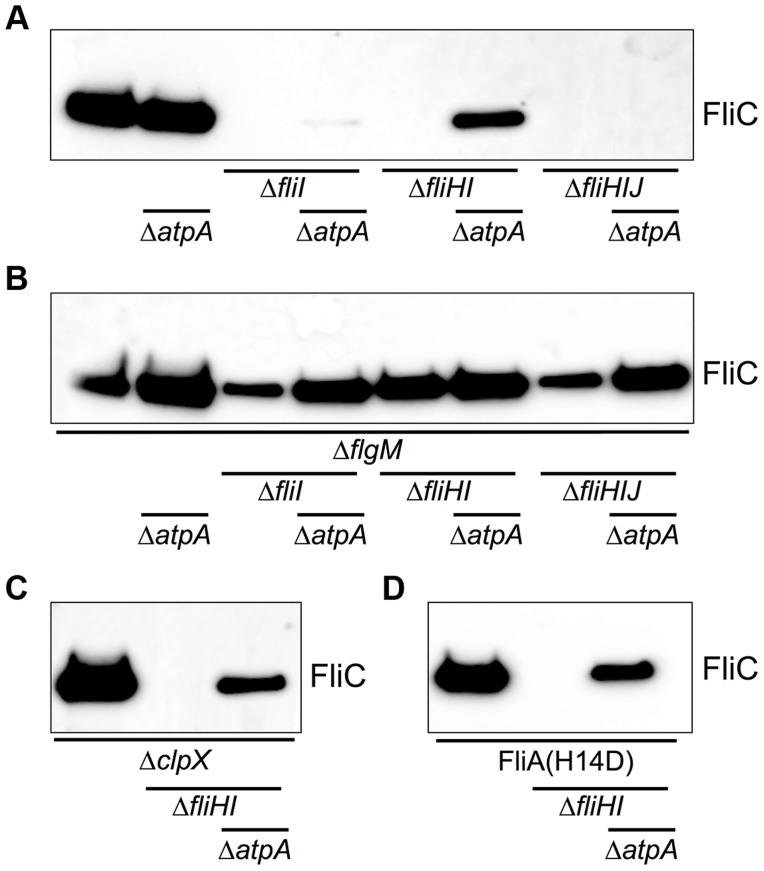
Flagellin protein secretion is restored in the absence of FliHIJ ATPase components by Δ*atpA*, *ΔflgM*, *ΔclpX*, and *fliA^H14D^* mutations. Secreted FliC flagellin protein was analyzed by anti-FliC immunostaining in the FliC-phase locked wildtype and *fliHIJ* mutant strains. (**A**) Wildtype and *fliHIJ* mutants in combination with Δ*atpA*. (**B**) Wildtype and *fliHIJ* mutants in combination with Δ*flgM* and Δ*atpA*. (**C**) Wildtype and *fliHI* deletion mutant in combination with Δ*clpX* and Δ*atpA*. (**D**) Wildtype and *fliHI* deletion mutant in combination with *fliA^H14D^* and Δ*atpA*.

**Figure 6:**
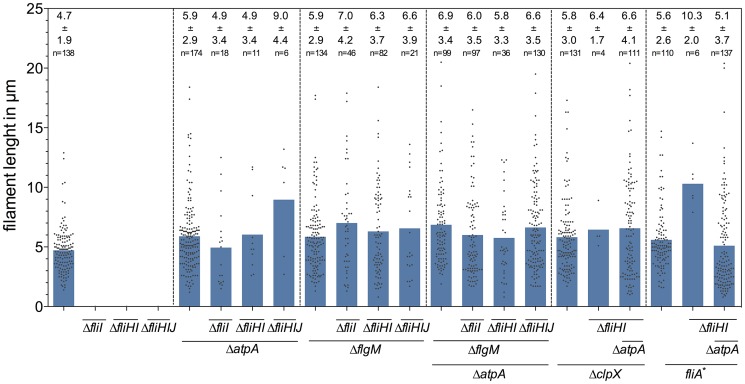
Lengths of flagellar filaments in *fliHIJ* mutants. Plot showing the lengths of individual flagellar filaments of the *fliHIJ* mutants visualized by anti-FliC immunostaining in Figure 3 and Figure 4. The average lengths of flagellar filaments +/− standard deviation and the number of measured filaments are presented in the upper part of the graph.

The cytoplasmic C-ring of the flagellum (composed of FliG, FliM, FliN) is essential for efficient localization of flagellar secretion substrates to the vicinity of the export gate by functioning as an affinity site for the localization of substrate/ATPase complexes. We have previously shown that a defect in the C-ring structure can be bypassed by providing an excess of secretion substrate [28]. Mutations in *flgM*, *clpX* and the *fliA* ^H14D^ (FlgM-bypass) allele result in increased secretion substrate levels. We tested the effects of increased substrate levels on secretion via the flagellar type-III secretion apparatus in the absence of the FliHIJ ATPase complex. In various *fliHIJ* mutant backgrounds null mutations of *flgM*, *clpX* or *fliA* ^H14D^ increased the frequency of bacteria able to produce at least one flagellum up to 35% of the population (Fig. 3C, Fig. 4). The deletion of *flgM* and *atpA* enhanced the frequency of flagellar assembly to a maximum of 74% of the population in the *fliHI* mutant background (Figs. 3C and 3D). The absence of the ATP synthase increased flagellar filament formation in the *clpX* or *fliA* ^H14D^ backgrounds to 55% and 36%, respectively (Figs. 4A and 4B). A significant increase in secreted flagellin was also observed in the *flgM*, *clpX* or *fliA* ^H14D^ mutant backgrounds under conditions when the PMF was apparently elevated by the *atpA* deletion (Fig. 5). We additionally measured the lengths of flagellar filaments visualized by flagellin immunostaining (Figs. 3 and 4) and observed that the average lengths of flagellar filaments of the various *fliHIJ* mutants is as long or longer than the wildtype filament lengths. The average lengths of filaments were also increased in an otherwise wildtype background if an excess of secretion substrates (e.g. by deleting the negative regulator *flgM*) was provided or the PMF was increased by deletion of the *atpA* subunit (Fig. 6).

In a complementary experiment, we tested the possibility that an increase in PMF (by deletion of *atpA*) or an excess of secretion substrates (by deletion of *flgM*) would allow for flagellar protein export in FlhA mutants where proton-flow through the export apparatus is blocked [33]. As shown in Fig. S6, no secretion of flagellin was detected under either condition, demonstrating that the proton flux through FlhA is essential for flagellar type-III secretion in contrast to ATP hydrolysis by the FliHIJ complex.

**Figure 7:**
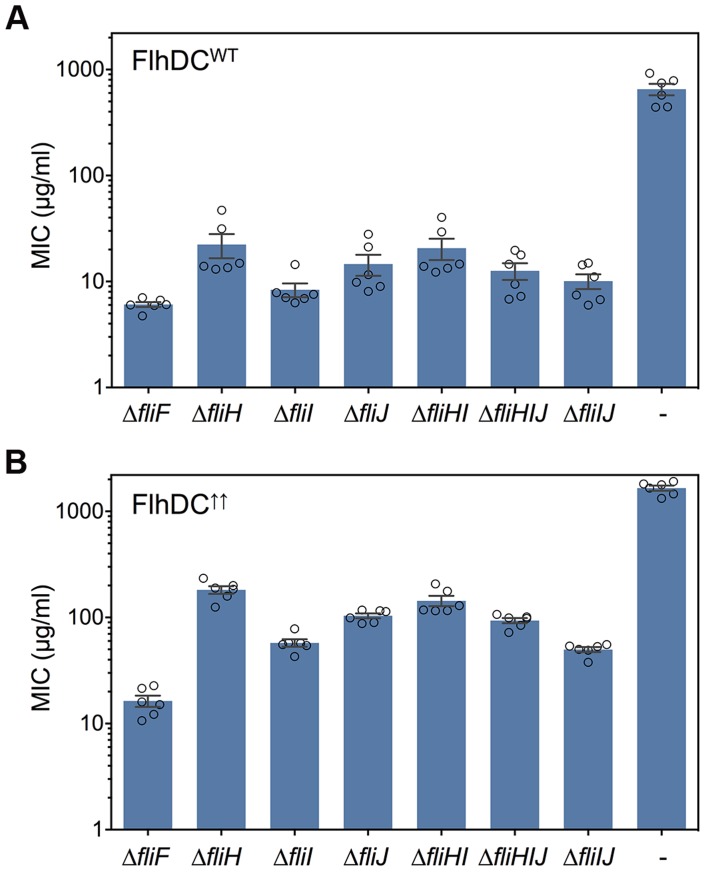
FliH functions as a negative regulator of type-III protein translocation. Export of FlgE-Bla fusion protein into the periplasm was analyzed in various *fliHIJ* deletion strains. All strains additionally harbored a deletion of the proximal rod genes (*ΔflgBC6557*) and the FlgE-Bla fusion protein under its native promoter (*flgE6569*::*bla*). (A) Minimal inhibitory concentration (MIC) values with flagellar genes expressed at normal levels. (B) Summary of MIC values with flagellar genes expressed at elevated levels due to a P*_flhD_* promoter-up mutation (P_flhD*_ = (P1+P4 -10 TATAAT)). The error bars represent the standard error of the mean (SEM) and biological replicates are shown as individual data points.

It has been suggested that the regulatory subunit of the ATPase complex, FliH, blocks the entrance gate of the secretion channel in the absence of its cognate FliI ATPase subunit [15], [16], [34]. In order to dissect the contribution of individual components of the FliHIJ complex to the type-III export process across the cytoplasmic membrane, we utilized a hook protein - β-lactamase based reporter system (FlgE-Bla). In the absence of the proximal rod subunits FlgB and FlgC, the FlgE-Bla fusion protein is secreted into the periplasm [28], [35], conferring quantifiable resistance to Ampicillin. We analyzed the export capability of various single and combination mutants of *fliH*, *fliI* and *fliJ* by determining the minimal inhibitory concentration (MIC) against Ampicillin. A deletion of the inner membrane scaffold protein, FliF, was used as a negative control and the MIC of the *fliF* mutant represents the basal level of Ampicillin resistance in this assay. Single deletion mutants of *fliH* and *fliJ* substantially increased the MIC about 3.5-fold compared to the *fliF* control strain (Fig. 7A). This is consistent with the previously reported leaky-motility phenotype of *fliH* and *fliJ* mutants [22], [26]. A deletion mutant of *fliI* also displayed increased MIC values, albeit significantly less than either *fliH* or *fliJ* null strains. Interestingly, if the *fliI* mutation was combined with either the *fliH* or *fliHJ*, the MIC values reached the level of the single *fliH* or *fliJ* null strains. Importantly, if only *fliH* was retained and both *fliI* and *fliJ* were deleted, the export capability was reduced to the level of the single *fliI* deletion. Under excess FlgE-Bla reporter construct conditions, secretion was also restored in the *fliI* single deletion mutant as MIC levels were 3.5-fold higher compared to the *fliF* control (Fig. 7B). The MIC values of the *fliH* and *fliJ* single mutants were 11 and 6.5-fold increased, respectively. Similar as under physiological FlgE-Bla substrate conditions, the MIC levels were increased 9 and 6-fold in *fliHI* or *fliHIJ* combinatory mutations to the levels of the single *fliH* and *fliJ* mutants. If only FliH was retained (Δ*fliIJ*), the MIC values were decreased to the level of the single *fliI* mutant. These results demonstrate that FliH functions as an inhibitor of the type-III export process. However, significant export can occur in the absence of both the negative regulator FliH and the ATPase FliI if excess substrate is provided, further adding evidence to our results that fT3SS can efficiently occur without ATP hydrolysis by FliI.

**Figure 8:**
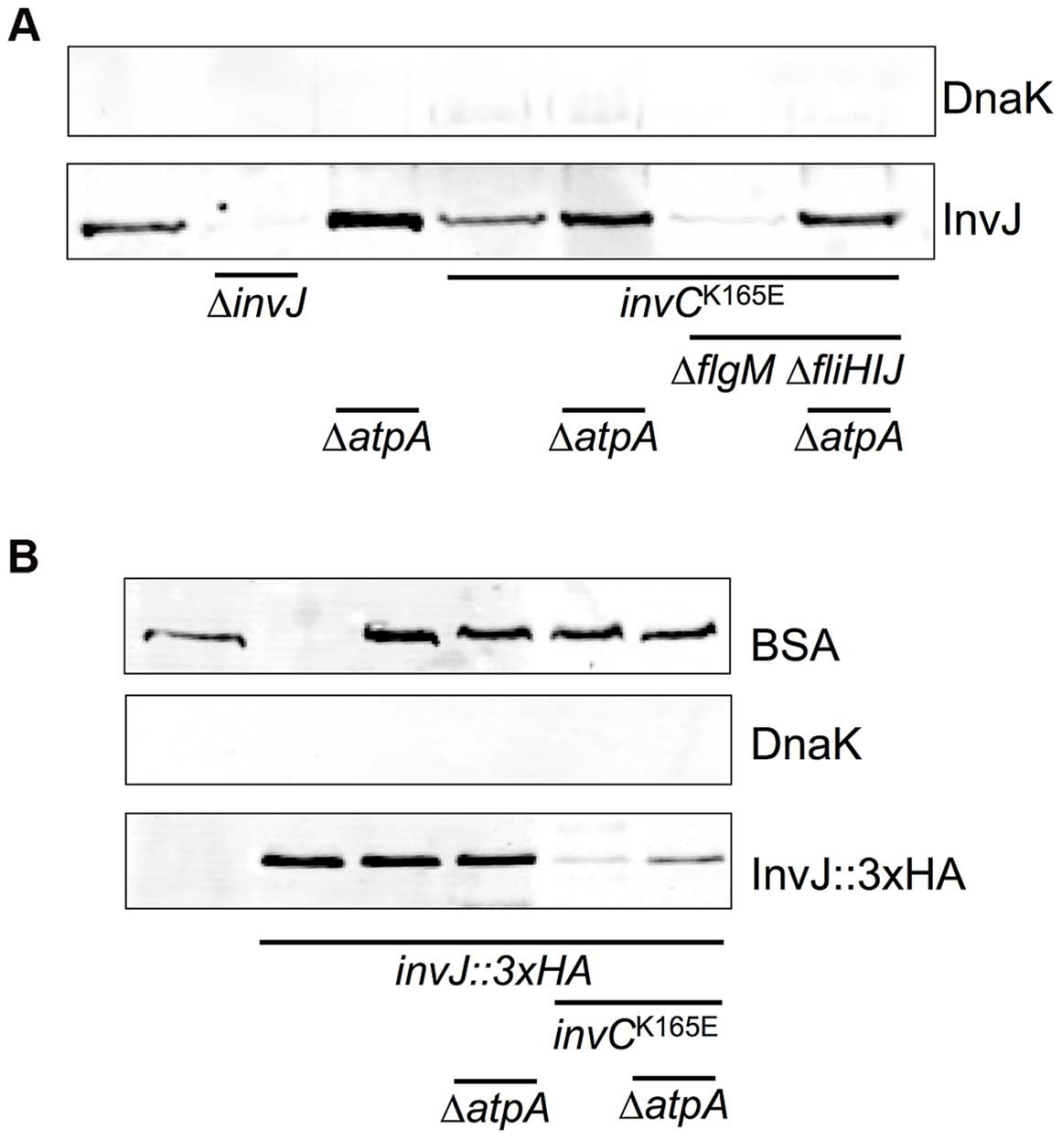
Protein secretion via the vT3SS in a catalytically-inactive ATPase mutant strain is rescued by deletion of *atpA*. Secretion of the Spi1 vT3SS substrate InvJ or a 3×HA tagged InvJ variant. (**A**) Secreted InvJ protein in the wildtype, Δ*invJ*, Δ*atpA*, the catalytically-inactive Spi1 ATPase *invC^K165E^*, *invC^K165E^* Δ*atpA*, *invC^K165E^* Δ*flgM*, and *invC^K165E^* Δ*flgM ΔfliHIJ ΔssaN ΔatpA* mutant strains. Detection of DnaK protein was included as a cell lysis control. (**B**) Levels of secreted InvJ::3×HA protein in the wildtype, Δ*invJ::3×HA*, Δ*invJ::3×HA* Δ*atpA*, Δ*invJ::3×HA invC^K165E^*, and Δ*invJ::3×HA invC^K165E^* Δ*atpA* mutant strains. 288 ng BSA was added to each supernatant fractions (except for lane 2) and served as a precipitation control. DnaK protein served as a cell lysis control.

The ATPase InvC is associated with the vT3SS encoded by Spi1 and essential for Spi1 vT3SS function and virulence [17], [36]. Previously, loss-of-function mutations in InvC have been isolated that specifically target membrane association, oligomerization and catalytic activity of the ATPase [37]. Here, we employed the catalytically-inactive InvC^K165E^ mutant to elucidate the role of ATPase activity in protein secretion via the Spi1 vT3SS. The InvC^K165E^ mutant strain secreted significantly less InvJ substrate protein, however the defect in secretion was bypassed by a null mutation in the *atpA* subunit of ATP synthase (Fig. 8A). Importantly, the fT3SS-associated ATPase FliI did not complement InvC function, as a *fliHIJ* deletion in the InvC^K165E^ Δ*atpA* mutant strain did not abolish InvJ secretion. Similar results were obtained using a 3×HA-tagged InvJ substrate protein (Fig. 8B).

## Discussion

The results demonstrate that ATPase activity is universally dispensable for protein export via bacterial type-III secretion systems. We propose that the function of the T3SS-associated ATPase complex is to deliver secretion substrates to the export apparatus and ensure efficient substrate unfolding, which was first reported by Akeda and Galán [17]. Either increased substrate production or increased PMF overcomes the requirement for the ATPase complex (Fig. S7). However, increased PMF or substrates do not bypass the requirement for proton flux through FlhA in contrast to the dispensability of ATP hydrolysis by the FliHIJ complex (Fig. S6).

In summary, our data demonstrate that efficient type-III protein export occurs in the absence of the ATP hydrolyzing components; therefore it is reasonable to assume that the export gate itself has a primary function in substrate unfolding and translocation. We speculate that the function of the ATPase complex in substrate unfolding and chaperone release is secondary to assure a highly efficient export process. Our data would suggest that chaperone release and unfolding occur inefficiently without ATPase activity, but is catalyzed by FliHIJ. Increasing PMF thus shifts the equilibrium of the spontaneous process.

The observation that export of a FlgE-Bla reporter construct was similarly increased in both the *fliHI* and *fliJ* mutant strains indicated that the FliHI and FliJ components of the ATPase complex acted in different pathways of the type-III export process. A possible scenario would be that FliJ – which interacts with FlhA of the export gate – activates the efficient Δψ-driven type-III protein export as suggested by Minamino et al. [21] (see also Fig. S7). FliH binds to both the ATPase FliI and the cytoplasmic C-ring, thereby localizing secretion substrates to the export gate for efficient unfolding by FliI prior to a Δψ or ΔpH-driven translocation process. In the absence of FliJ, the inner membrane export apparatus can still function as a less-efficient ΔpH-driven protein-proton antiporter and the secretion process is facilitated by substrate unfolding via the FliHI complex. The non-essential substrate unfolding is not occurring in a *fliHI* mutant strain, however the export gate operates in the highly-efficient Δψ export mode, which might be able to energize substrate unfolding as well.

The switch from early to late (flagellin-like) substrate secretion is a bottleneck in the process of flagellar bioassembly. The importance of this step in flagellar bioassembly was evidenced by the fact that we observed assembly of single flagellar filaments of wildtype lengths and longer in our *fliHIJ* deletion mutants (Fig. 6). It has been demonstrated before that the inner membrane ring complex, the C-ring and integral membrane components of the fT3SS assemble normally in the absence of the *fliHIJ* complex [38], [39]. Thus, the *fliHIJ* mutants analyzed here presumably assemble a physiological number of secretion system complexes. Only upon completion of the hook-basal-body structure, the secretion apparatus undergoes its secretion substrate specificity switch. In the absence of the FliHIJ complex, flagellar protein export is strongly impaired and thus HBB completion and the switch to filament-type secretion is a rare event. However, there appears to exist a mechanism for localization of the available late flagellar substrates to the single hook-basal-body that is in a late-export-competent state since we observed filament lengths that were as long or longer than the wildtype in our *fliHIJ* mutants (Fig. 6). An increase in PMF then likely assists in efficient export of late secretion substrates and thereby overcomes the requirement of the FliHIJ ATPase complex for late substrate secretion – similar to the related scenario where excess substrates bypass the requirement of the FliHIJ ATPase complex.

Our findings also have important implications for the evolution of the bacterial flagellum and type-III secretion systems. As mentioned above, the cytoplasmic components of the type-III secretion system share strong homology to components of the F_O_F_1_ ATP synthase and it has been proposed that the flagellum was derived from a proto F_O_F_1_ ATP synthase where ATP hydrolysis might have energized the export process. The present results would indicate that a proto F_1_-ATPase was added to a primordial type-III export system (which might have been proton-powered) with the evolutionary benefit of facilitating the export process. This made the bacterial type-III secretion apparatus to the highly efficient protein-export system of the contemporary flagellum and injectisome.

In summary, type-III protein export is a process of great complexity and involves a substantial number of checkpoints to ensure the correct order of export. A reasonable, general proposal for the export mechanism of bacterial type-III secretion systems is that the respective ATPase complexes bind to substrate proteins, shuttle the substrates to the base of the growing structure, energize chaperone release and substrate unfolding in an ATP-dependent manner and present the substrate to the membrane-embedded export apparatus components for efficient PMF-dependent secretion.

## Materials and Methods

### Bacterial strains, plasmids and media

All bacterial strains used in this study are listed in Table 1. Cells were grown in either lysogeny broth (LB) [40] or TB broth (1% Tryptone and 0.5% NaCl). Motility agar was prepared as described before [28]. The generalized transducing phage of *Salmonella enterica* servovar Typhimurium P22 *HT105*/*1 int-201* was used in all transductional crosses [41].

**Table 1:** List of *Salmonella enterica* servovar Typhimurium LT2 strains used in this study.

Strain number	Relevant genotype	Reference
TH437	wildtype	J. Roth
TH6232	*Δhin-5717::FRT*	[47]
TH8208	Δhin-5717::FRT fliA*5225(H14D)	Lab collection
TH8419	*ΔinvJ::FKF*	Lab collection
TH9949	*flgE6569::bla ΔflgBC655*	[35]
TH11801	*ΔatpA::tetRA*	[15]
TH12465	*flgE6569*::*bla* Δ*flgBC655 ΔfliF7387*	This study
TH12472	*flgE6569*::*bla* Δ*flgBC655 ΔfliH7394*	This study
TH12473	*flgE6569*::*bla* Δ*flgBC655 ΔfliI7395*	This study
TH12474	*flgE6569*::*bla* Δ*flgBC655 ΔfliJ7396*	This study
TH12475	*flgE6569*::*bla* Δ*flgBC655 ΔfliHI7397*	This study
TH12476	*flgE6569*::*bla* Δ*flgBC655 ΔfliHIJ7398*	This study
TH12477	*flgE6569*::*bla* Δ*flgBC655 ΔfliIJ7399*	This study
TH13867	*Δhin-5717::FRT ΔfliI7364*	This study
TH13868	*Δhin-5717::FRT ΔfliHI7366*	This study
TH13869	*Δhin-5717::FRT ΔfliHIJ7367*	This study
TH14002	*Δhin-5717::FRT clpX1::*Tn*10d*Cm *ΔfliHI7366*	This study
TH14009	*Δhin-5717::FRT ΔflgM5628::FRT ΔfliI7364*	This study
TH14010	*Δhin-5717::FRT ΔflgM5628::FRT ΔfliHI7366*	This study
TH14011	*Δhin-5717::FRT ΔflgM5628::FRT ΔfliHIJ7367*	This study
TH14017	Δhin-5717::FRT fliA*5225 (H14D)	This study
TH14018	Δhin-5717::FRT fliA*5225 (H14D) *ΔfliHI7366*	This study
TH14019	Δhin-5717::FRT fliA*5225 (H14D) *ΔfliHIJ7367*	This study
TH14129	*Δhin-5717::FRT ΔfliHI7366 ΔclpX*	This study
TH14130	*Δhin-5717::FRT clpX1::*Tn*10d*Cm *ΔfliHI7366 atp* (Mot+)	This study
TH14184	*ΔfliHI7366 Δhin-5717::FRT ΔclpX ΔatpA::tetRA*	This study
TH14260	*ΔflgM5628::FRT ΔfliHI7366 Δhin-5717::FRT ΔatpA::tetRA*	This study
TH14261	fliA*5225 (H14D) *ΔfliHI7366 Δhin-5717::FRT ΔatpA::tetRA*	This study
TH14292	*ΔfliHI7366 Δhin-5717::FRT ΔatpA::tetRA*	This study
TH14826	*Δhin-5717::FCF ΔflgM5628::FRT*	This study
TH15426	*flgE6569::bla ΔflgBC655 PflhD7793* (P1+P4 -10 TATAAT)	This study
TH15427	*flgE6569::bla ΔflgBC655 PflhD7793* (P1+P4 -10 TATAAT) *ΔfliF7387*	This study
TH15430	*flgE6569::bla ΔflgBC655 PflhD7793* (P1+P4 -10 TATAAT) *ΔfliI7395*	This study
TH15431	*flgE6569::bla ΔflgBC655 PflhD7793* (P1+P4 -10 TATAAT) *ΔfliHIJ7398*	This study
TH15925	*flgE6569::bla ΔflgBC655 PflhD7793* (P1+P4 -10 TATAAT) *ΔfliJ7396*	This study
TH16031	*flgE6569::bla ΔflgBC655 PflhD7793* (P1+P4 -10 TATAAT) *ΔfliH7394*	This study
TH16032	*flgE6569::bla ΔflgBC655 PflhD7793* (P1+P4 -10 TATAAT) *ΔfliHI7397*	This study
TH20154	*invC314* (K165E)	This study
TH20637	*invC314 ΔatpA::tetRA*	This study
TH20708	*invC314* Δ*flgM5628*::FRT Δ*fliHIJ7367* Δ*ssaN110*	This study
TH20709	*invC314* Δ*flgM5628*::FRT Δ*ssaN110* Δ*fliHIJ7367 ΔatpA*::*tetRA*	This study
TH20714	*invJ320*::*3×HA* (SAGASA linker on both sides of 3×HA; between aa140 and 141)	This study
TH20750	*invJ320*::*3×HA ΔatpA*::*tetRA*	This study
TH20830	*invC314 invJ321*::*3×HA* (SAGASA linker on both sides of 3×HA; between aa140 and 141)	This study
TH20831	*invC314 invJ322::3×HA* (SAGASA linker on both sides of 3×HA; between aa140 and 141) *ΔflgM5628::FRT ΔfliHIJ7367 ΔssaN110*	This study
TH20832	*invC314 invJ321::3×HA ΔatpA::tetRA*	This study
TH20833	*invC314 invJ322::3×HA ΔflgM5628::FRT ΔfliHIJ7367 ΔssaN110 ΔatpA::tetRA*	This study
EM404	*Δhin-5717::FRT ΔflgM5628::FRT*	This study
EM405	*Δhin-5717::FRT ΔatpA::tetRA*	This study
EM406	*Δhin-5717::FRT ΔfliI7364 ΔatpA::tetRA*	This study
EM407	*Δhin-5717::FRT ΔfliHIJ7367 ΔatpA::tetRA*	This study
EM408	*Δhin-5717::FRT ΔflgM5628::FRT ΔfliI7364 ΔatpA::tetRA*	This study
EM409	*Δhin-5717::FRT ΔflgM5628::FRT ΔfliHIJ7367 ΔatpA::tetRA*	This study
EM415	*Δhin-5717::FRT ΔflgM5628::FRT ΔatpA::tetRA*	This study
EM417	*Δhin-5717::FRT ΔclpX*	This study
EM1236	*flgE6569::bla ΔflgBC655 PflhD7793* (P1+P4 -10 TATAAT) *ΔfliIJ7399*	This study
EM1959	*Δhin-5717::FRT flhA22345* (D208A) *ΔatpA::tetRA*	This study
EM1960	*Δhin-5717::FRT flhA22346* (D208E) *ΔatpA::tetRA*	This study
EM1961	*Δhin-5717::FRT flhA22347* (D208K) *ΔatpA::tetRA*	This study
EM2037	*Δhin-5717::FRT flhA22345* (D208A) *ΔflgM5628::FKF*	This study
EM2038	*Δhin-5717::FRT flhA22346* (D208E) *ΔflgM5628::FKF*	This study
EM2039	*Δhin-5717::FRT flhA22347* (D208K) *ΔflgM5628::FKF*	This study

### Protein secretion assay

Secretion of FliC and InvJ into the culture supernatant was analyzed as described previously [32], [42]. For analysis of InvJ secretion, cultures were grown under Spi1-inducing conditions (high-osmolarity (0.3M NaCl, final) and low-oxygen (without agitation)) [43]. Secreted proteins were separated from the cellular fraction and precipitated using a final concentration of 10% Trichloroacetic acid (TCA). Protein levels of cellular and secreted fractions were analyzed by SDS polyacrylamide gel electrophoresis and immunoblotting. Antibodies against FliC (rabbit), InvJ (rabbit, kind gift of Sam Miller, University of Washington), hemaglutinin (mouse, Pierce), bovine serum albumin (mouse, Pierce) and DnaK (mouse, Abcam) were used for detection.

### Minimal inhibitory concentration assay (FlgE-Bla secretion)

The minimal inhibitory concentration against ampicillin resulting from the export of hook-β-lactamase (FlgE-Bla) fusion proteins was analyzed as described by Lee et al. [35] with minor modifications. Briefly, cultures were grown at 37°C in a 96-well plates in LB media to mid-log phase. An aliquot was transferred in LB media supplemented with increasing concentrations of fresh ampicillin. Cells were grown for 4.5 hours at 37°C in a shaking incubator before absorbance was measured at OD_600_. The minimal inhibitory concentration values were determined as follows using R for Mac OS X and a modified version of package ‘stinepack’. Relative growth values were calculated by normalization of the measured OD_600_ values of any ampicillin (Amp) concentration to the zero Amp control. Next, a Stineman interpolation was used for a smooth curve fit of the relative growth values and root finding was performed to determine the MIC by calculating the interception with a threshold set at 5% of residual growth of the zero Amp control.

### Fluorescent microscopy

Fluorescent microscopy analysis was performed as described before [32]. Briefly, logarithmically growing bacteria were applied to a poly-L-lysine treated coverslip well. Bacterial cells were fixed by addition of final 2% formaldehyde and 0.2% glutaraldehyde. Flagella were stained using polyclonal anti-FliC antibodies (rabbit) and anti-rabbit conjugated Alexa Fluor 488 secondary antibodies (Invitrogen). DNA staining was performed using Hoechst (Invitrogen) and cell membranes were stained using FM-64 (0.5 mg ml^−1^, Invitrogen). Images were collected with optical Z sections every 100 nm using an Applied Precision optical sectioning microscope and deconvolved using softWoRx v.3.4.2 (Applied Precision). The pixel data of individual Z sections of the deconvolved images were projected on a single plane using the Z-project tool (settings: maximal intensity) of ImageJ v.1.6.0 [44].

### Free swimming assay

Bacterial cultures were grown in TB media at 37°C and aliquots were mixed with TB media containing 10% (final) Ficoll 400 (Sigma) and applied to a microscopic chamber consisting of a microscope slide and coverslip spaced by two strips of double sided adhesive tape for free swimming speed analysis. Free-swimming bacterial cells were observed close to the coverslip surface and at least two 30-second movies from different field of views were recorded. Movies were imported into ImageJ v.1.6.0 and the trajectories of several hundred cells were determined by the “2D ParticleTracker” ImageJ plug-in (http://www.mosaic.ethz.ch/Downloads/ParticleTracker, [45]). The median velocities of at least 100 cells were calculated using a custom-made [R] script as described by [46].

## Supporting Information

Figure S1 Mutations in *atp* locus restore motility of a *fliHI* mutant strain. (A) Representative soft agar motility plate of motility suppressors of the non-motile *fliHI* mutant. Exemplary motility swarms of the wildtype TH6232 (WT), the parental *fliHI* strains TH13868 (Δ*fliHI*) and TH14002 (Δ*fliHI clpX*), and the originally isolated suppressor mutant in the *atp* locus TH14130 (Δ*fliHI clpX atp*) are shown. The mutation in the *atp* locus restored motility of the *fliHI* deletion strain. Motility plates were incubated at 37°C for 4.5 hours before imaging. The *clpX* mutation alone does not increase motility after 4.5 hours incubation but note that the original suppressor mutants in *clpXP* were isolated after overnight incubation. (B) Quantified relative motility of the *fliHIJ* suppressor strains. The diameter of the motility swarm relative to the wildtype was measured after 4.5 hours incubation. Biological replicates are shown as individual data points.
(TIFF)

Figure S2 Quantification of motility of *fliHIJ* mutant strains. Representative images used for quantification of the swimming motility assay shown in Figure 2B. Swimming motility plates containing 0.3% agar were incubated for 4.5 hours at 37°C before imaging.
(TIFF)

Figure S3 Growth curves of the wildtype and Δ*atpA* mutant. Growth of the wildtype strain TH437 (WT) and Δ*atpA* strain TH11801 (Δ*atpA*) in lysogeny broth (A) and tryptone broth (B). An overnight culture was diluted 1∶100 in LB or TB medium in a 96-well plate and incubated at 37°C. Growth was monitored by measuring the OD_600_ every 15 minutes.
(TIFF)

Figure S4 Swimming speed of the *ΔatpA* mutant strain. Velocities of individual cells of the wildtype (TH6232) and the Δ*atpA* mutant strain (EM405) were determined in 30 min intervals throughout the growth curve starting at an optical density of approximately 0.4 (time = 0). Velocities of at least 100 individual cells are depicted as box diagrams with whiskers according to the Tukey method. The increase of the optical density (OD_600_) of both wildtype and Δ*atpA* mutant is shown in the inlet.
(TIFF)

Figure S5 Induction of PMF-draining TetA tetracycline/proton antiporter suppresses the restored motility of *fliHIJ ΔatpA* mutant strains. (A) Quantified relative motility of the wildtype (TH6232) and various *fliHIJ atpA* mutant strains Δ*fliI* (TH13867), Δ*fliI ΔatpA* (EM406), Δ*fliHI* (TH13868), Δ*fliHI ΔatpA* (TH14292), Δ*fliHIJ* (TH13869) and Δ*fliHIJ ΔatpA* (EM407) in the presence of the *tetA* inducer anhydrotetracycline. The *atpA* gene in strains EM406, TH14292 and EM407 was deleted using a *tetRA* resistance cassette. Expression of *tetA* was induced by addition of 1 µg/ml anhydrotetracycline (AnTc). Biological replicates are shown as individual data points. Data were analyzed by the Student's *t* test. Stars indicate significantly different motility (ns, non significant). (B) Representative soft agar motility plates after 4.5 hours incubation at 37°C in the presence of anhydrotetracycline.
(TIFF)

Figure S6 Mutations in Δ*atpA* or Δ*flgM* do not rescue flagellar protein export of FlhA Asp-208 mutants. Secreted FliC flagellin protein was analyzed by anti-FliC immunostaining in FliC-phase locked strains. The charged residue Asp-208 of FlhA has been previously implicated in proton flow through the export apparatus [33]. Increased levels of flagellar substrates and PMF were provided by a deletion of *atpA* or *flgM*, respectively. (**A**) Secretion of FliC under increased PMF conditions. Strains harbored a Δ*atpA* deletion and the wildtype *flhA* allele (EM405) or the *flhA* point mutations D208A (EM1959), D208E (EM1960) and D208K (EM1961). (**B**) Secretion of FliC under elevated substrate conditions. Strains harbored a Δ*flgM* deletion and the wildtype *flhA* allele (TH14826) or the *flhA* point mutations D208A (EM2037), D208E (EM2038) and D208K (EM2039).
(TIFF)

Figure S7 Model for the flagellar type-III protein export process. A schematic model of the flagellar type-III protein secretion process is presented as described in the text. The FliJ component of the ATPase complex interacts with FlhA of the membrane-embedded export apparatus and activates the efficient Δψ-driven type-III protein export [21]. Upper part: Under wildtype conditions, the FliHIJ ATPase complex binds to substrate proteins, shuttles the substrates to the base of the export apparatus, energizes chaperone release and substrate unfolding in an ATP-dependent manner and presents the substrate to the membrane-embedded export apparatus components for efficient proton motive force (PMF)-dependent secretion. The Δψ component of the PMF is utilized for the export process. Middle part of the figure: in a Δ*fliH* mutant, localization of secretion substrates to the export gate by binding of FliH to the C-ring is prevented. In a *fliHI* mutant strain, the non-essential substrate unfolding is not occurring. However, the membrane-embedded export apparatus operates in the highly-efficient Δψ export mode due to the presence of FliJ. Lower part of the figure: In the absence of FliJ (Δ*fliIJ* or Δ*fliJ*), the membrane components of the export apparatus can still function as a less-efficient ΔpH-driven protein-proton antiporter and the secretion process is facilitated by substrate unfolding via the FliHI complex in the Δ*fliJ* mutant strain.
(TIFF)
