# Supplementary figures and images for: ATPase-Independent Type-III Protein Secretion in Salmonella enterica
Source: PLoS Genet. 2014 Nov 13;10(11):e1004800. doi: 10.1371/journal.pgen.1004800 (PMC4230889; doi:10.1371/journal.pgen.1004800)

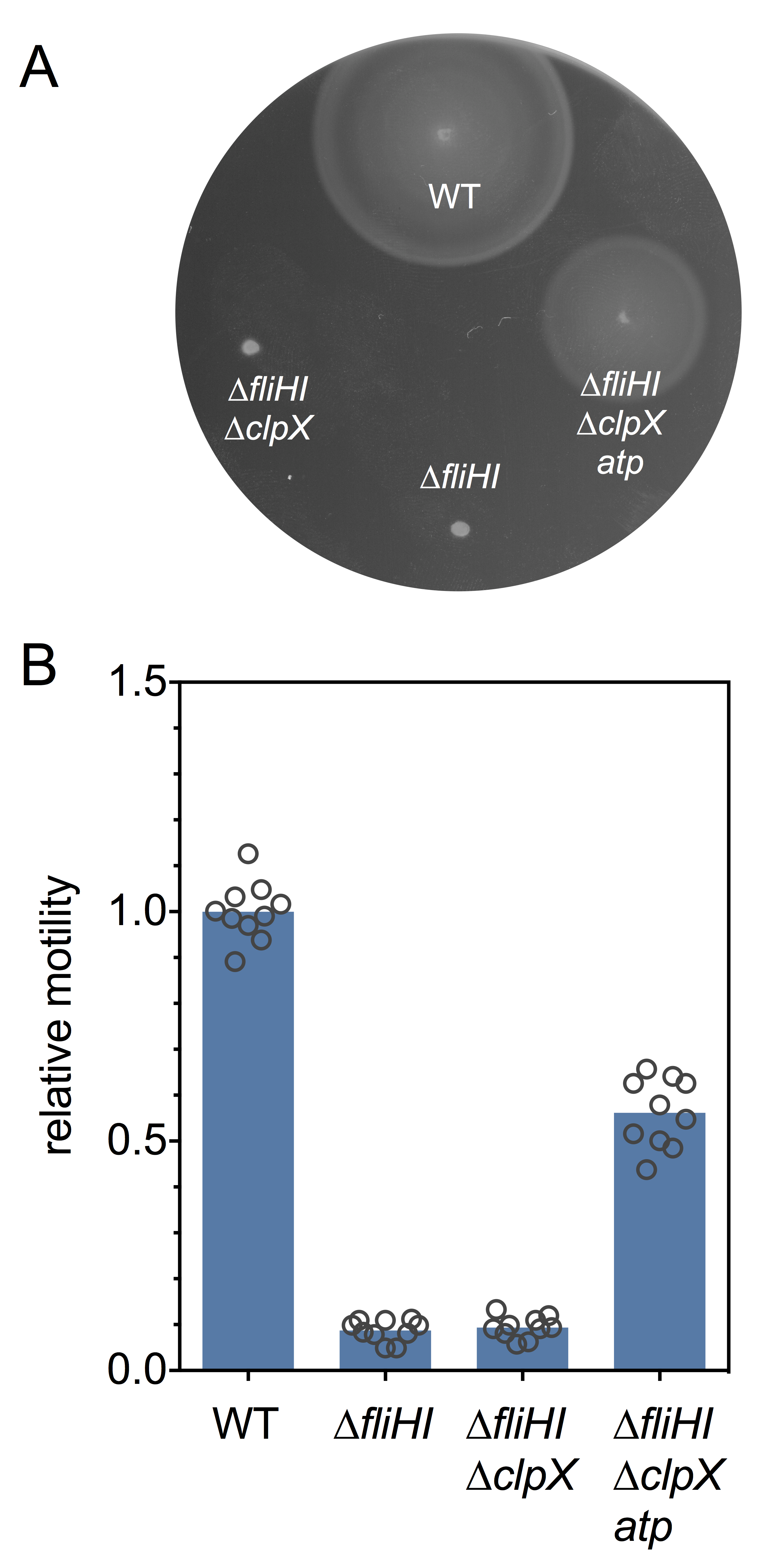

Supplement: Figure S1 — Mutations in atp locus restore motility of a fliHI mutant strain. (A) Representative soft agar motility plate of motility suppressors of the non-motile fliHI mutant. Exemplary motility swarms of the wildtype TH6232 (WT), the parental fliHI strains TH13868 (ΔfliHI) and TH14002 (ΔfliHI clpX), and the originally isolated suppressor mutant in the atp locus TH14130 (ΔfliHI clpX atp) are shown. The mutation in the atp locus restored motility of the fliHI deletion strain. Motility plates were incubated at 37°C for 4.5 hours before imaging. The clpX mutation alone does not increase motility after 4.5 hours incubation but note that the original suppressor mutants in clpXP were isolated after overnight incubation. (B) Quantified relative motility of the fliHIJ suppressor strains. The diameter of the motility swarm relative to the wildtype was measured after 4.5 hours incubation. Biological replicates are shown as individual data points. (TIFF) [file pgen.1004800.s001.tiff]

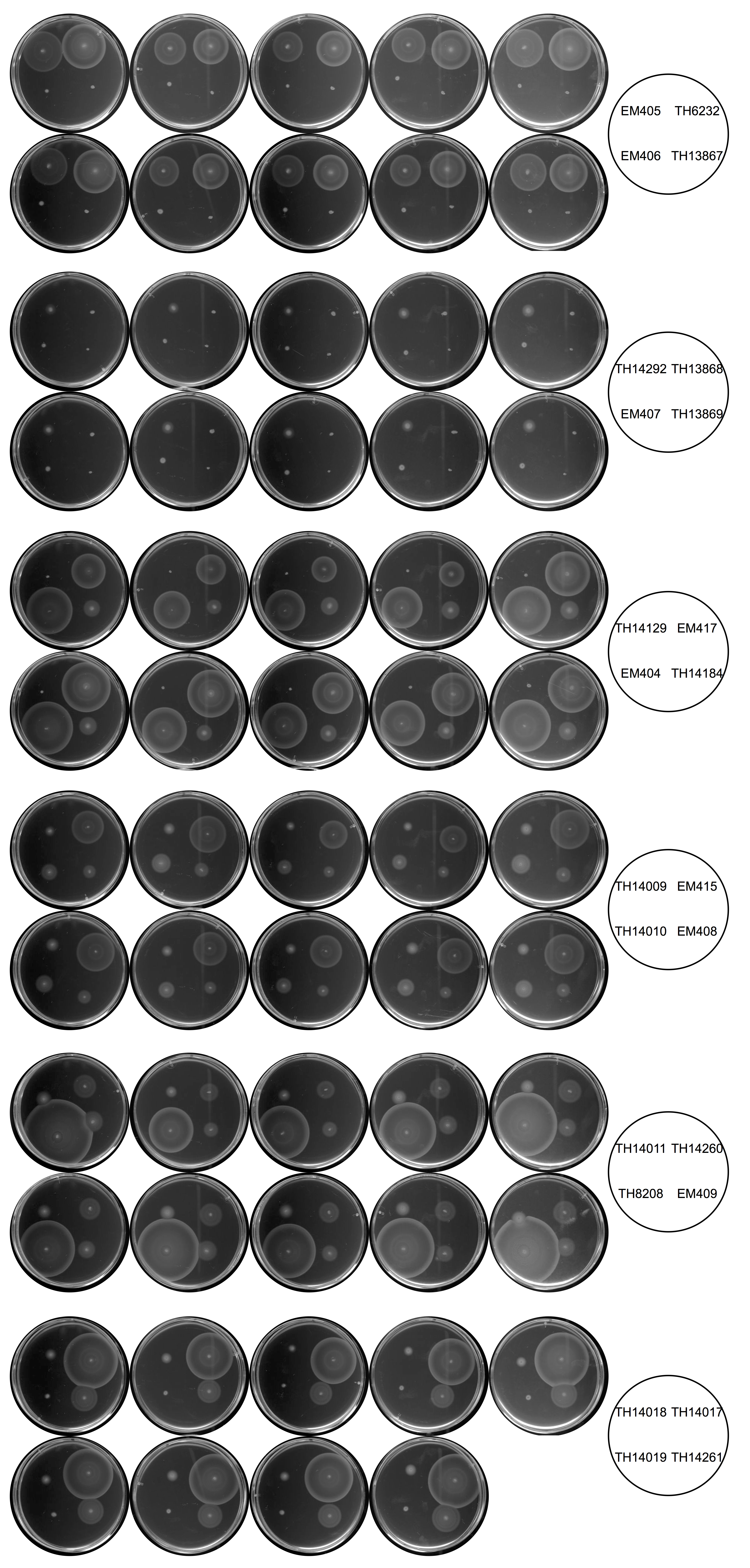

Supplement: Figure S2 — Quantification of motility of fliHIJ mutant strains. Representative images used for quantification of the swimming motility assay shown in Figure 2B. Swimming motility plates containing 0.3% agar were incubated for 4.5 hours at 37°C before imaging. (TIFF) [file pgen.1004800.s002.tiff]

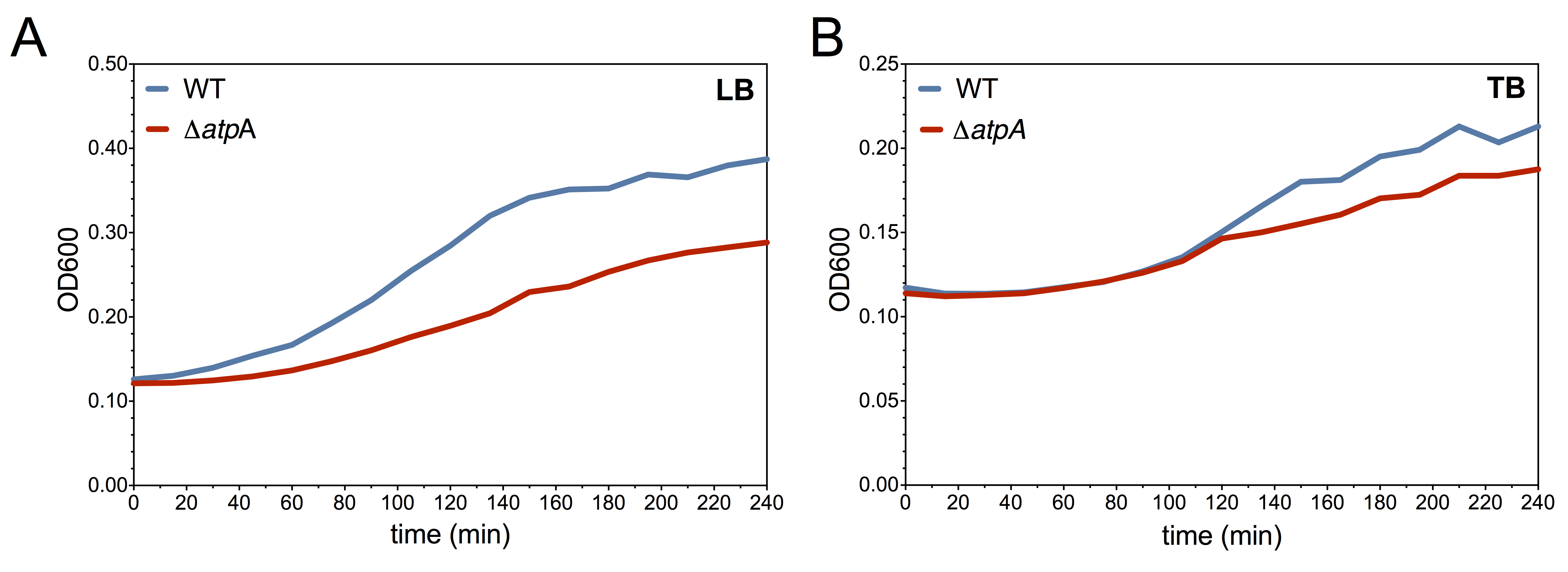

Supplement: Figure S3 — Growth curves of the wildtype and ΔatpA mutant. Growth of the wildtype strain TH437 (WT) and ΔatpA strain TH11801 (ΔatpA) in lysogeny broth (A) and tryptone broth (B). An overnight culture was diluted 1∶100 in LB or TB medium in a 96-well plate and incubated at 37°C. Growth was monitored by measuring the OD600 every 15 minutes. (TIFF) [file pgen.1004800.s003.tiff]

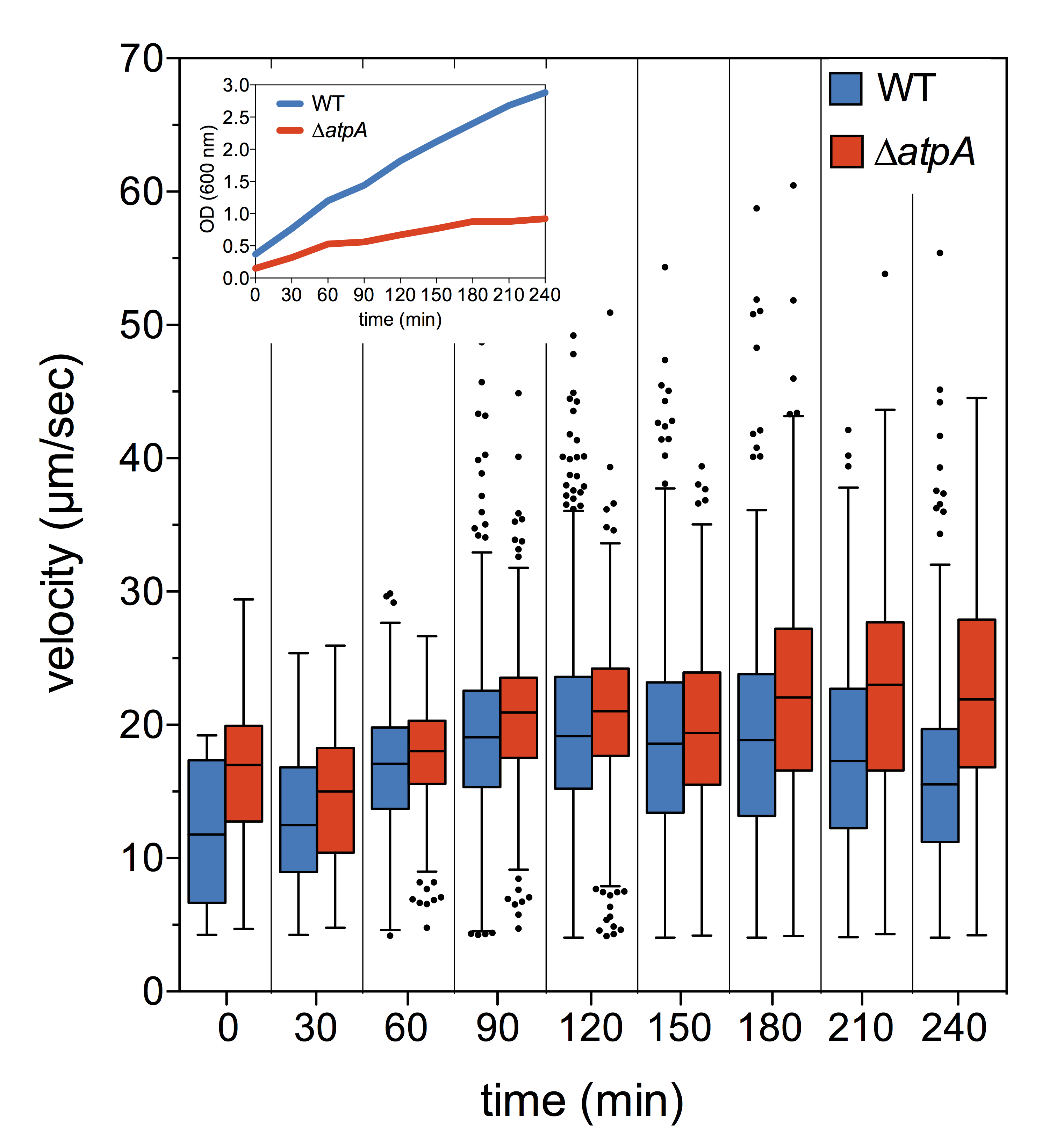

Supplement: Figure S4 — Swimming speed of the ΔatpA mutant strain. Velocities of individual cells of the wildtype (TH6232) and the ΔatpA mutant strain (EM405) were determined in 30 min intervals throughout the growth curve starting at an optical density of approximately 0.4 (time = 0). Velocities of at least 100 individual cells are depicted as box diagrams with whiskers according to the Tukey method. The increase of the optical density (OD600) of both wildtype and ΔatpA mutant is shown in the inlet. (TIFF) [file pgen.1004800.s004.tiff]

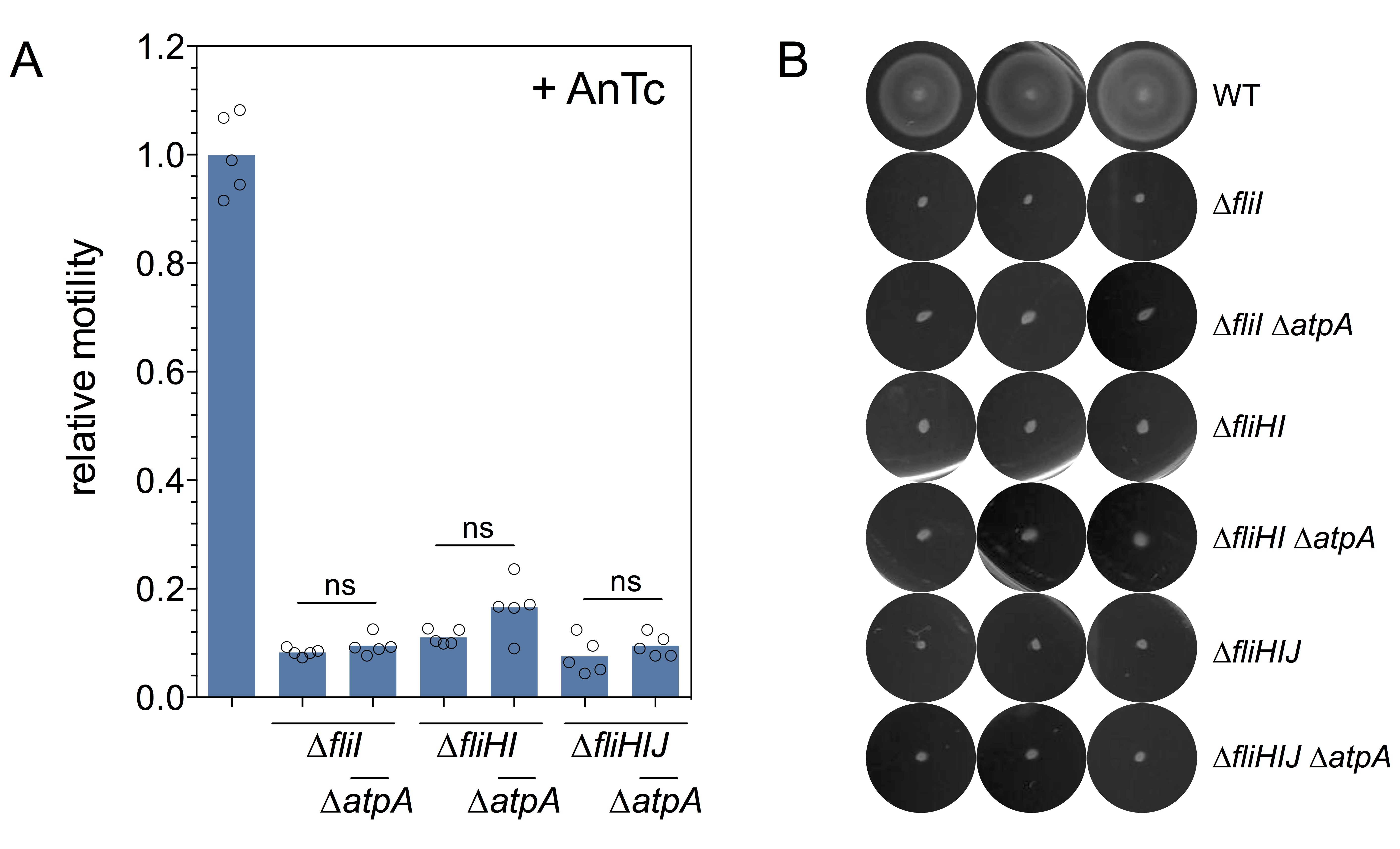

Supplement: Figure S5 — Induction of PMF-draining TetA tetracycline/proton antiporter suppresses the restored motility of fliHIJ ΔatpA mutant strains. (A) Quantified relative motility of the wildtype (TH6232) and various fliHIJ atpA mutant strains ΔfliI (TH13867), ΔfliI ΔatpA (EM406), ΔfliHI (TH13868), ΔfliHI ΔatpA (TH14292), ΔfliHIJ (TH13869) and ΔfliHIJ ΔatpA (EM407) in the presence of the tetA inducer anhydrotetracycline. The atpA gene in strains EM406, TH14292 and EM407 was deleted using a tetRA resistance cassette. Expression of tetA was induced by addition of 1 µg/ml anhydrotetracycline (AnTc). Biological replicates are shown as individual data points. Data were analyzed by the Student's t test. Stars indicate significantly different motility (ns, non significant). (B) Representative soft agar motility plates after 4.5 hours incubation at 37°C in the presence of anhydrotetracycline. (TIFF) [file pgen.1004800.s005.tiff]

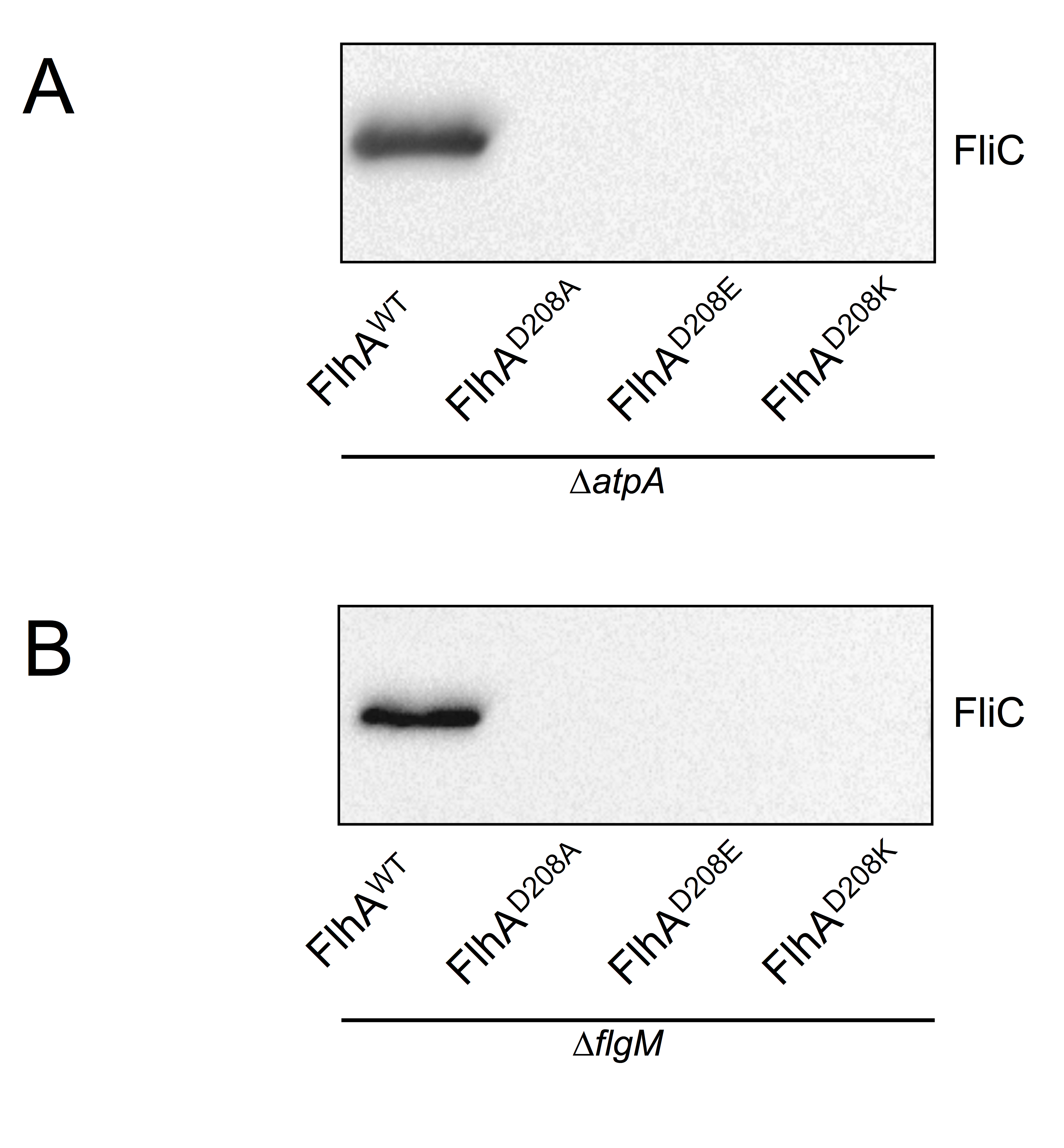

Supplement: Figure S6 — Mutations in ΔatpA or ΔflgM do not rescue flagellar protein export of FlhA Asp-208 mutants. Secreted FliC flagellin protein was analyzed by anti-FliC immunostaining in FliC-phase locked strains. The charged residue Asp-208 of FlhA has been previously implicated in proton flow through the export apparatus [33]. Increased levels of flagellar substrates and PMF were provided by a deletion of atpA or flgM, respectively. (A) Secretion of FliC under increased PMF conditions. Strains harbored a ΔatpA deletion and the wildtype flhA allele (EM405) or the flhA point mutations D208A (EM1959), D208E (EM1960) and D208K (EM1961). (B) Secretion of FliC under elevated substrate conditions. Strains harbored a ΔflgM deletion and the wildtype flhA allele (TH14826) or the flhA point mutations D208A (EM2037), D208E (EM2038) and D208K (EM2039). (TIFF) [file pgen.1004800.s006.tiff]

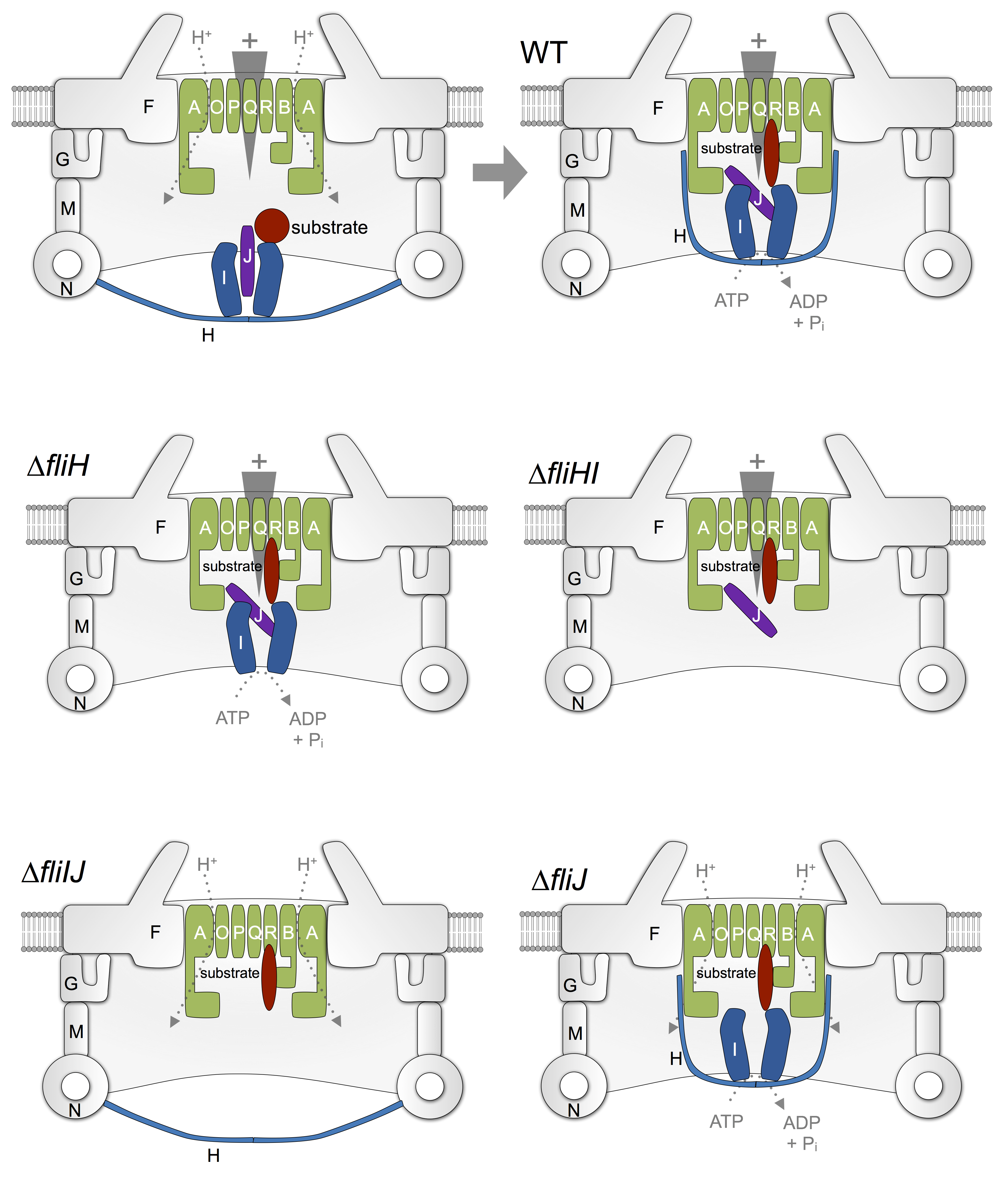

Supplement: Figure S7 — Model for the flagellar type-III protein export process. A schematic model of the flagellar type-III protein secretion process is presented as described in the text. The FliJ component of the ATPase complex interacts with FlhA of the membrane-embedded export apparatus and activates the efficient Δψ-driven type-III protein export [21]. Upper part: Under wildtype conditions, the FliHIJ ATPase complex binds to substrate proteins, shuttles the substrates to the base of the export apparatus, energizes chaperone release and substrate unfolding in an ATP-dependent manner and presents the substrate to the membrane-embedded export apparatus components for efficient proton motive force (PMF)-dependent secretion. The Δψ component of the PMF is utilized for the export process. Middle part of the figure: in a ΔfliH mutant, localization of secretion substrates to the export gate by binding of FliH to the C-ring is prevented. In a fliHI mutant strain, the non-essential substrate unfolding is not occurring. However, the membrane-embedded export apparatus operates in the highly-efficient Δψ export mode due to the presence of FliJ. Lower part of the figure: In the absence of FliJ (ΔfliIJ or ΔfliJ), the membrane components of the export apparatus can still function as a less-efficient ΔpH-driven protein-proton antiporter and the secretion process is facilitated by substrate unfolding via the FliHI complex in the ΔfliJ mutant strain. (TIFF) [file pgen.1004800.s007.tiff]
